# Inhibition of farnesyl pyrophosphate synthase improves pressure overload induced chronic cardiac remodeling

**DOI:** 10.1038/srep39186

**Published:** 2016-12-23

**Authors:** Chen-Ze Zhao, Xu-Ming Zhao, Jian Yang, Yun Mou, Bin Chen, Huan-Dong Wu, Dong-Pu Dai, Jie Ding, Shen-Jiang Hu

**Affiliations:** 1 From the Institute of Cardiology, The First Affiliated Hospital, School of Medicine, Zhejiang University, Hangzhou, PR China; 2 Department of Ultrasound, The First Affiliated Hospital, School of Medicine, Zhejiang University, Hangzhou, PR China; 3 Department of Cardiology, Hangzhou First Municipal Hospital and Affiliated Hangzhou Hospital of Nanjing Medical University, Hangzhou, China

## Abstract

Farnesyl pyrophosphate synthase (FPPS) is a key enzyme in the mevalonate pathway. In our previous studies, we find that inhibition of FPPS attenuates angiotensin II-induced cardiac hypertrophy and fibrosis by suppressing RhoA while FPPS and Ras are up-regulated in pressure overload rats. In this study, we evaluate the effects and mechanisms of FPPS inhibition in pressure overload mice. Male FPPS-small interfering RNA (SiRNA) transgenic (Tg) mice and non-transgenic littermate control (NLC) were randomly divided into suprarenal abdominal aortic constriction (AAC) group and sham operation group. 12 weeks following AAC, mice were sacrificed by cervical dislocation. Histological and echocardiographic assessments showed that inhibition of FPPS improved chronic cardiac remodeling which was induced by AAC. The reductions of Ras farnesylation and GTP-Ras, as well as their downstream extracellular signal-related kinases 1/2 (ERK1/2) expression were observed in the heart of Tg-AAC mice compared with NLC-AAC mice, along with the reduction of fetal gene expression. We provide here important experimental evidence that inhibition of FPPS improves AAC induced chronic cardiac remodeling and fibrosis by the reduction of farnesylated Ras and the downregulation of Ras-ERK1/2 pathway.

Heart failure is one of the leading causes of morbidity and mortality worldwide. Abnormal cardiac remodeling plays a vital role in the pathogenesis of chronic heart failure^1^. In response to chronic pressure overload, the heart initially increases ventricle wall and interventricular septum dimensions to normalize the diastolic and systolic function^2^. If the sustained stimuli exceeds that of the compensatory capacity of the heart, subsequent degradation of the ECM and alterations of the collagen network will progressively result in alterations of left ventricular morphology and function, which later on turn into heart failure^3^. There is also an increase in the expression of embryonic genes, including the brain natriuretic peptide (BNP) and β-myosin heavy chain (β-MHC).

Farnesyl pyrophosphate synthase (FPPS) is a key enzyme in the mevalonate pathway. FPPS catalyzes the formation of geranyl pyrophosphate (GPP) and farnesyl pyrophosphate (FPP)^4^. FPP is an important substrate not only in cholesterol and coenzyme Q biosynthesis, but also in the farnesylation of small GTPases, such as Ras,. For Ras to function as signal transducer, it has to be farnesylated near the C-terminus by farnesyltransferase (FTase) and bind to the plasma membrane^5^,^6^. Ras hyperactivity is closely associated with cardiac remodeling in the cardiomyocytes^7^,^8^,^9^.

Our previous studies have reported that inhibition of FPPS attenuates angiotensin II-induced cardiac hypertrophy and fibrosis by deceasing RhoA activity^10^ while overexpression of FPPS induces cardiac hypertrophy and dysfunction by increasing RhoA expression^11^. Interestingly, the upregulation of Ras preceded the increase of RhoA in pressure overload induced cardiac hypertrophy^12^. Moreover, inhibition of farnesyltransferase improved cardiac remodeling in spontaneously hypertensive rats by reducing Ras activity^13^. Therefore, a decreasing effect of Ras might be more effective than that of RhoA in pressure overload mouse model. In this study, FPPS small interfering RNA transgenic mice^14^ and their non-transgenic littermate control which subjected to abdominal aortic constriction or sham operation were used to further investigate the effect of FPPS in pressure overload.

## Results

### Hearts showed hypertrophy following AAC

**Figure 1:**
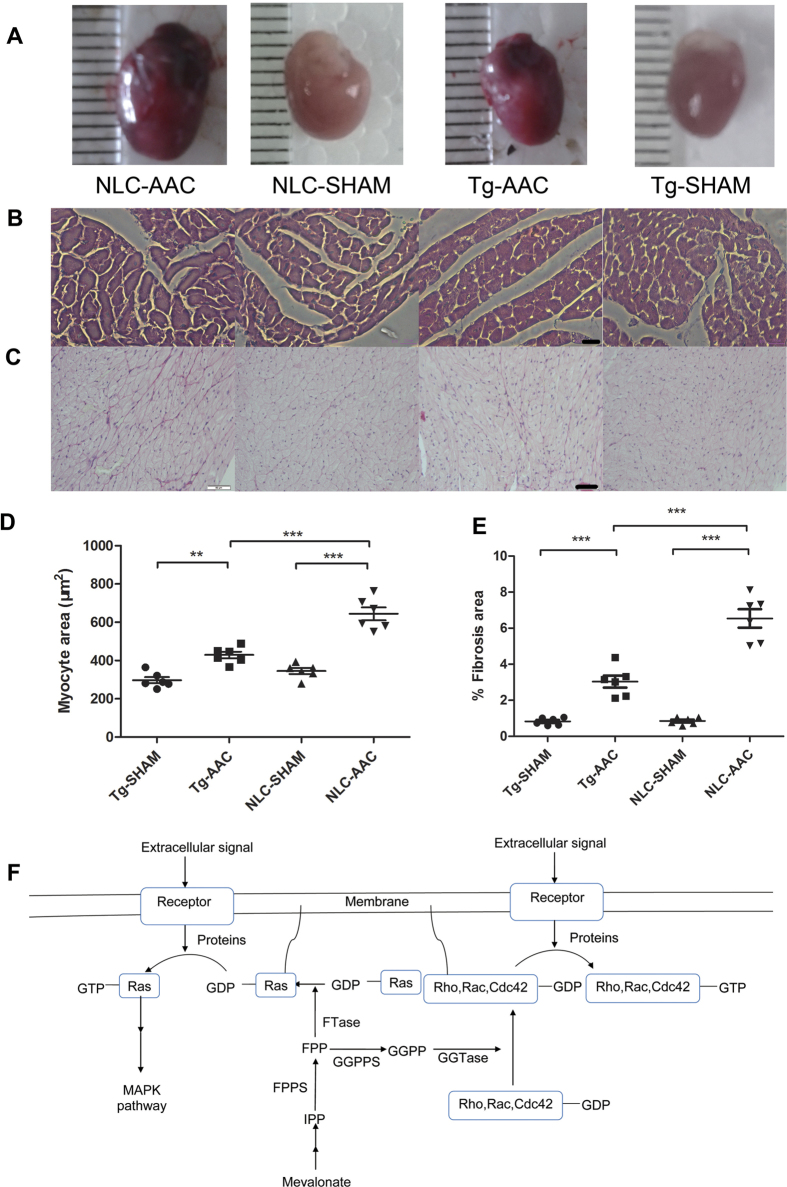
Characterization of cardiac phenotypes in AAC and Tg mice (**A**) Gross morphology of hearts from sham/AAC and NLC/Tg mice. (**B**) Histological assessment of cardiac sections staining sham/AAC and NLC/Tg mice by hematoxylin and eosin staining. Scale bar: 20 μm (**C**) Histological assessment of cardiac sections staining sham/AAC and NLC/Tg mice by Picrosirius Red staining. Scale bar: 50 μm (**D**) Quantification of the average area of cardiomyocyte. (**E**) Quantification of the fibrosis area (red) from Picrosirius Red-stained sections. (**F**) Model of small GTP-binding proteins activation. NLC, non-transgenic littermate control; Tg, transgenic; AAC, abdominal aortic constriction; IPP, isopentenyl pyrophosphate; FPP, farnesyl pyrophosphate; GGPP, geranylgeranyl pyrophosphate; FPPS, farnesyl pyrophosphate synthase; GGPPS, geranylgeranyl pyrophosphate synthase; FTase, farnesyltransferase; GGTase, geranylgeranyltransferase. MAPK, mitogen-activated protein kinase ***P < 0.001; **P < 0.01.

**Figure 2:**
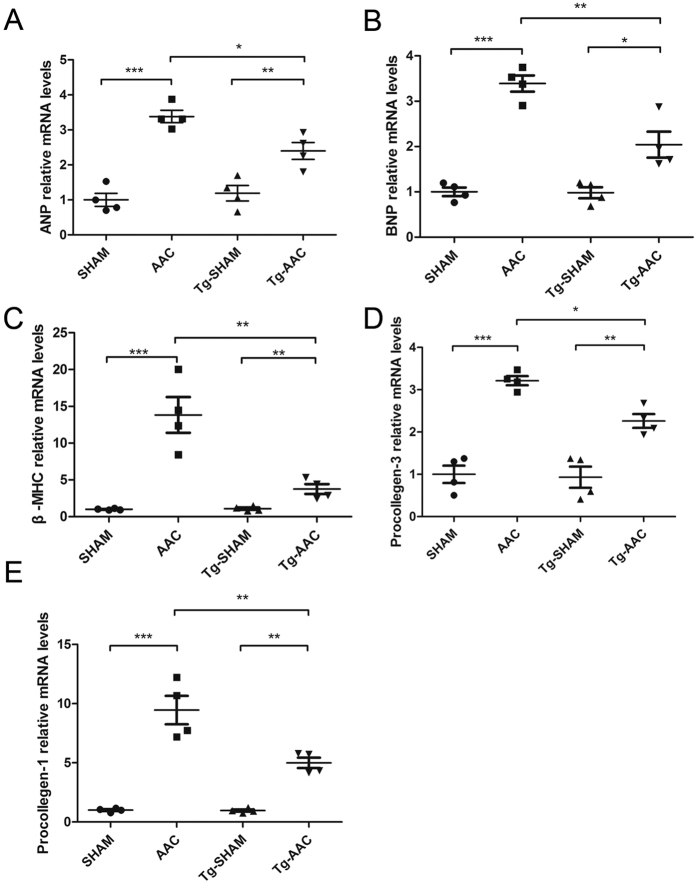
Quantification of hypertrophy- and fibrosis-associated mRNA levels in 4 groups’ hearts. GAPDH was the loading control. NLC, non-transgenic littermate control; Tg, transgenic; AAC, abdominal aortic constriction; ANP, atrial natriuretic peptide; BNP, brain natriuretic peptide; β-MHC, β-myosin heavy chain. ***P < 0.001; **P < 0.01; *P < 0.05.

**Figure 3:**
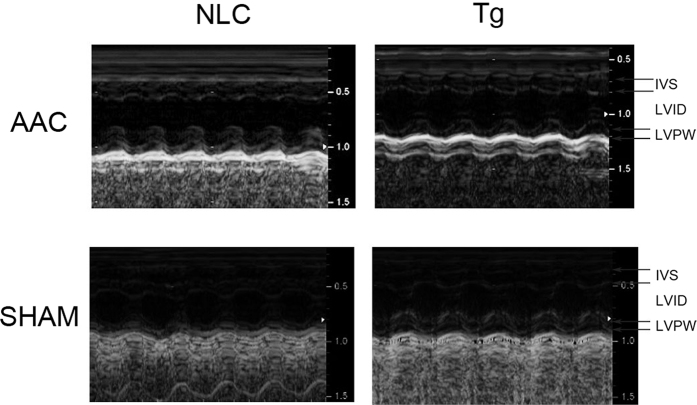
M-mode pictures from the echocardiography. NLC, non-transgenic littermate control; Tg, transgenic; AAC, abdominal aortic constriction. IVS, interventricular septum thickness; LVPW, left ventricular posterior wall thickness; LVID, left ventricular internal dimension.

12 weeks following AAC, the total heart weights of NLC-AAC group were enlarged approximately 20% compared with that in NLC-sham group, so that heart weight/body weight ratios or heart weight/tibia length ratios were increased at the similar level (Table 1). Microscopically, the areas of myocardial cell surface after AAC were clearly enlarged (Fig. 1B,D). As expected, the expression of heart failure markers, atrial natriuretic peptide (ANP), brain natriuretic peptide (BNP) and β-myosin heavy chain (β-MHC) were all increased as accessed by qPCR (Fig. 2A–C). Echocardiography showed that the interventricular septum thickness in end-diastole (IVSd) and left ventricular posterior wall thickness in end-diastole (LVPWd) were significantly increased in the mice after AAC, with enlarged left ventricular internal dimension in end-diastole (LVIDd) and left ventricular internal dimension in end-systole (LVIDs) and decreased ejection fractions (EF) (Table 2, Fig. 3). All of above indicated that the mice after AAC were suffering heart hypertrophy.

### FPPS interfering improved AAC induced cardiac hypertrophy

Hearts from FPPS-transgenic mice were macroscopically indistinguishable from those of NLC mice as previous described^14^. Importantly, the enlargement of heart weight/body weight ratios and myocardial cell surface area was cut approximately 40% in tg-AAC mice compared with that in NLC-AAC mice (p < 0.01). The average area of myocardial cell surface was also diminished in the Tg-AAC hearts (Fig. 1B,D). This might owe much to the decrease of LVIDd and LVIDs and the similar thickness of the left ventricular wall. To assess the left ventricular contractility, ejection fraction (EF) and fractional shortening (FS) were calculated. The transgenic effect partially improved the worse contractile function (Table 2, Fig. 3). Additionally, the ANP, BNP and β-MHC levels were all decreased in Tg-AAC mice (Fig. 2A–C). These data indicated that FPPS interfering improved AAC induced cardiac hypertrophy.

### FPPS interfering attenuated interstitial fibrosis

Certain marker genes were analysed for cardiac fibrosis. The cardiac mRNA levels of procollagen I and III raised incredibly in the NLC mice subject to AAC (Fig. 2D,E). Similarly, in Tg-AAC group, the increases were partially suppressed. Histopathological analysis was in accord with the changes in gene expression. Image-Pro 6.0 was used to calculate the fibrosis area. Picrosirius Red staining presented 5–8% interstitial fibrosis area in NLC-AAC group, but 2–5% in the Tg-AAC group (Fig. 1C,E).

### Expression of key enzymes in isoprenoid metabolism

**Figure 4:**
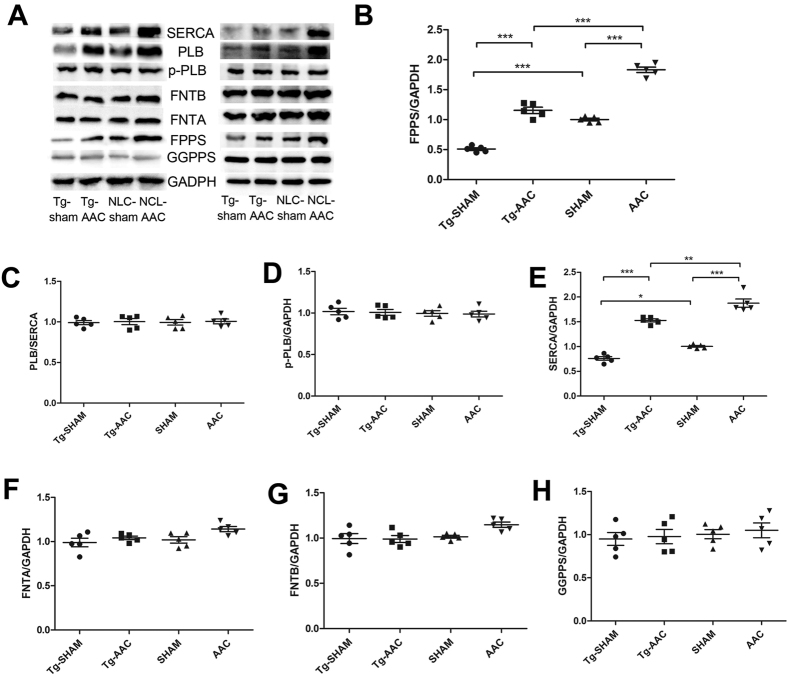
Expression of key enzymes in the mevalonate pathway and calcium regulation. Equal loading was validated by the expression of GAPDH. NLC, non-transgenic littermate control; Tg, transgenic; AAC, abdominal aortic constriction; FPPS, farnesyl pyrophosphate synthase; GGPPS, geranylgeranyl pyrophosphate synthase; FNTA, farnesyltransferase-α FNTB, farnesyltransferase-β; SERCA, Sarcoplasmic reticulum Ca-ATPase; PLB, Phospholamban ***P < 0.001; **P < 0.01; *P < 0.05.

As expected, FPPS was significantly upregulated after AAC but downregulated in transgenic mice (Fig. 4A,B). FPPS is a branch point enzyme in mevalonate pathway. Altering FPPS expression might affect the other downstream enzymes. Geranylgeranyl pyrophosphate synthase (GGPPS) did not differ after Tg or AAC (Fig. 4A,H). The expressions of farnesyltransferase-α (FNTA) and farnesyltransferase-β (FNTB) were a bit higher in NLC-AAC mice than that in sham operation mice while little changes were discovered in Tg-AAC mice (Fig. 4F,G).

### SERCA is decreased in Tg mice

Sarcoplasmic reticulum Ca-ATPase 2a (SERCA2a) is primarily expressed in the heart and is the mediator of calcium uptake by the sarcoplasmic reticulum, initiating relaxation^15^. Phospholamban (PLB) is the reversible inhibitor of SERCA2a and cardiac function. Phosphorylation of PLB relieves the inhibition by blocking the binding interaction with SERCA2a^16^. Interestingly, SERCA2a was notably decreased in transgenic mice but it was remarkably increased following AAC. The ratios of PLB/SERCA2a and the expressions of p-PLB were equal in four groups to keep the intracellular Ca^2+^ homeostasis (Fig. 4A,C,D,E).

### The activities of small GTP-binding protein

**Figure 5:**
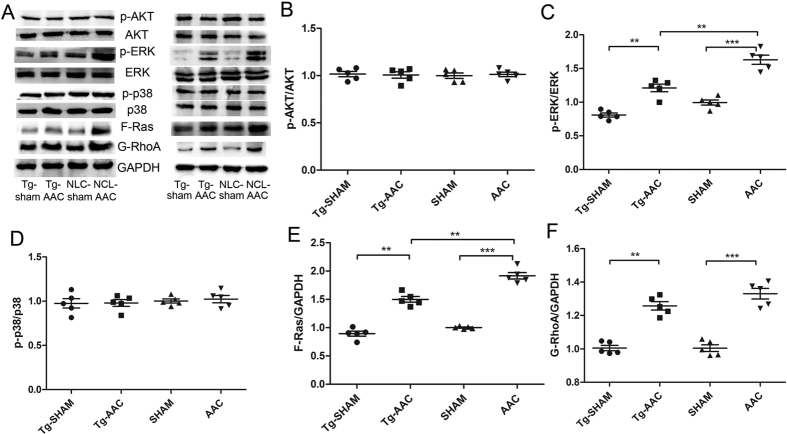
Expression of small G-proteins in membrane and their downstream factors. Equal loading was validated by the expression of GAPDH. NLC, non-transgenic littermate control; Tg, transgenic; AAC, abdominal aortic constriction; F-Ras, farnesylated Ras; G-RhoA, geranylgeranylated RhoA. ***P < 0.001; **P < 0.01; *P < 0.05.

**Figure 6:**
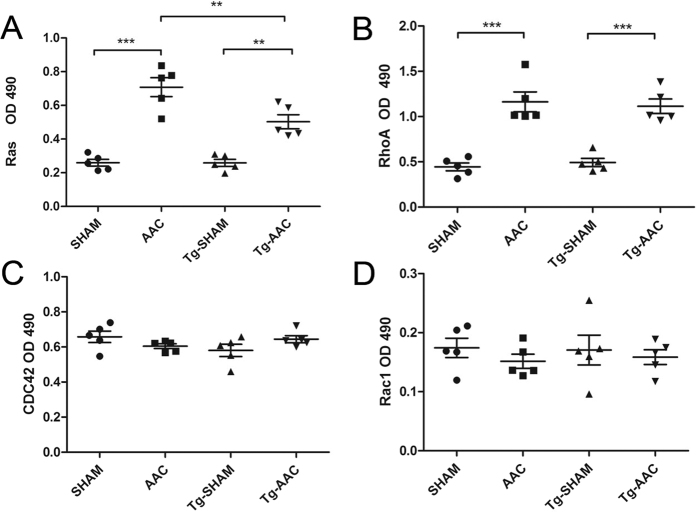
Quantification of GTP-binding small G-proteins. NLC, non-transgenic littermate control; Tg, transgenic; AAC, abdominal aortic constriction; ***P < 0.001; **P < 0.01; *P < 0.05.

Protein farnesylation and geranylgeranylation are necessary for the location of small GTPases such as Ras and Rho, independently^6^. The farnesyl moiety of Ras is necessary for their binding to the membranes, and the change of farnesylated Ras was in keeping with the change of FPPS. Significant increases of farnesylated Ras, GTP-Ras and extracellular signal-regulated kinase 1/2 (ERK1/2) were found in the AAC group compared with that in the sham group, whereas the increases were partially prevented by FPPS interfering (Figs 5A and 6A). Some other downstream effectors of Ras, such as p-38 and AKT, did not alter in these four groups (Fig. 5A,B,D). The Rho/Rac/Cdc42 family was located on the plasma membrane by geranylgeranylation. The expressions of GTP-RhoA and the RhoA binding to the cytomembrane were increased 12 weeks following AAC while FPPS interfering did not statistically suppress the increase (Figs 6B and 5A,F). Neither Rac1 nor Cdc42 activation was changed after AAC (Fig. 6C,D). These results indicated that inhibition of FPPS diminished the upregulation of farnesylated Ras, GTP-Ras and ERK1/2, but did not suppress the increase of RhoA 12 weeks following AAC.

## Discussion

In this study, we evaluated the effects and the possible mechanisms of FPPS interfering on AAC-induced cardiac remodeling *in vivo*. This is the first demonstration that inhibition of FPPS improved AAC-induced cardiac remodeling by the reduction of Ras farnesylation and activation, which subsequently triggered Ras-ERK1/2 signaling pathway.

Mevalonate pathway plays a vital role in cell survival. All of 3-hydroxy-3-methyl glutaryl coenzyme A reductase (HMGR), FTase and GGTase liver-specific knockout mice had been reported severe hepatic damage and most of them died in 3 months^17^,^18^. Xu *et al*. had showed that cardiac-specific GGPPS deletion led to spontaneous cardiac hypertrophy and subsequent heart failure and adult death due to a high ratio of FPPS/GGPPS^19^. FPPS knockout should break off the synthesis of FPP and interrupt the prenylation of proteins. Hence, we inferred that the mice with cardiac-specific knockout of FPPS might have a defective cardiac structure or function, which lead to embryonic death or spontaneous adult death.

Abdominal aortic constriction has been widely used to induce cardiac hypertrophy and heart failure in mouse model. The constricted level was highly depended on the external diameter of needle. Ji and colleagues demonstrated that heterozygous SERCA2a knockout mice exhibiting depressed contractile function, survived without signs of heart disease^20^. Therefore, We speculated that the Tg mice which had a decreased SERCA2a level suffered a higher lethality in acute heart failure. In the beginning, a 29- gauge needle was chosen for the destination of aortic binding but all of the Tg-AAC mice were unable to regain consciousness. Not until a 26-gauge needle was performed, a similar death rate was observed between Tg-AAC group and NLC-AAC group. 12 following AAC, the hearts showed a similar remodeling as previous reported^21^.

The elevated blood pressure plays an important role in the pathologic process of cardiac remodeling. In this experiment, the pressures of tail artery were shown that no statistic difference was found among four groups (Table 1). It may be concluded that inhitibion of FPPS didn’t significantly affect the systolic pressure comparing the blood pressure of Tg mice with that of NLC mice (118.4 ± 4.1 vs 119.3 ± 4.0 mmHg). However, we didn’t measure the blood pressure proximal to constriction site for our technical reason. There are limitations in measuring blood pressure via the tail, as this method did not evaluate changes in pressure gradient proximal to the abdominal constriction (i.e. close to the constriction in the aorta), hence it cannot be established whether the FPPS effects on hypertrophy and fibrosis are due to cardiac or vascular (afterload) effects. So it is also reasonable to hypothesize that arterial pressure proximal to the constriction have slight reductions after inhibition of FPPS, and the reductions improve cardiac remodeling. Consquencely, the reductions in hypertrophy and fibrosis observed are secondary to the effects directly in the heart and/or the reductions in magnitude of pressure gain with the coarctation procedure due to FPPS inhibition. The mechanism between these two possible effects still needs futher study.

It is well known that heart failure is associated with diminished SERCA2a expression^15^,^22^. In our study, the protein level of SERCA2a was found to be increased after AAC. This contradiction can be due to the fact that the hearts were in compensatory stage with an adequate ejection fraction. Upregulation of SERCA2a strengthened the cardiac contractility in acute cardiac dysfunction. In addition, an increased expression of SERCA2a could also be found in the early stage of metabolic syndrome^23^ and exercise^24^. However, the surplus energy demand created by SERCA2a may be harmful to the heart. In this case, the reduction of SERCA2a might be a better adaption for the heart. Hence, an increased expression of PLB was found to normalize the activity of SERCA2a after the acute cardiac dysfunction.

Ras proteins are crucially important molecules by inducing a large variety of extracellular signals. FPP, which synthetized by FPPS, is the very substrate of farnesylation. Farnesylation is necessary for their binding to membranes and their activation of downstream effectors. Ras activates a kinase cascade involving Raf, mitogen-activated protein kinases (MAPK) and extracellular signal-regulated kinase (ERK) phosphorylation, which in turn induces cell proliferation that leads to pathogenic myocardial hypertrophy and subsequent heart failure^2^,^25^. Transgenic mice expressing the constitutively active Ha-Ras-Val-12 were demonstrated ventricular hypertrophy^8^. Mouse models of inducible cardiac overexpression of Ras-Val-12 had shown pathogenic myocardial hypertrophy while discontinuation of Ras activity after induction of pathogenic myocardial hypertrophy led to resolution of cardiomyopathy^9^. Recently, Ramos-Kuri *et al*. reported that dominant negative Ras attenuates pathological ventricular remodeling in pressure overload cardiac hypertrophy^7^. Importantly, carabin protected against cardiac hypertrophy by blocking Ras and ERK activation via its Ras-GAP domain^21^. In the present study, knockdown of FPPS attenuated the active Ras and suppressed the expression of ANP, BNP and β-MHC and ultimately improved cardiac remodeling.

Ras-ERK1/2 alteration was also found in cardiac fibrosis. Hyperactivation of Ras and downstream pathways lead to marked cardiac hypertrophy, progressive cardiomyopathy, and fibrosis in the adult neurofibromin knockout mice^26^. Fibronectin, collagen I accumulation, overall interstitial fibrosis and the myofibroblast population were lower in the H-Ras knockout mice than in the wild-type mice^27^. Inhibiting ERK1/2 activity represses cell proliferation and collagen gene expression in activated cardiac fibroblasts^28^. Li *et al*. also reported that inhibition of ERK1/2 attenuated TGF-β1 expression, Smad2/3 nuclear accumulation, and periostin expression^29^. Accordingly, we indicated that inhibition of FPPS improved cardiac fibrosis by the diminished active Ras.

The Rho family of small GTP-binding proteins, including the Rho, Rac, and Cdc42 subfamilies, regulates cytoskeletal function in many aspects^30^. Geranylgeranylation with GGPP, which is synthesized from FPP by GGPPS, is necessary for the activation of RhoA. We had demonstrated that overexpression of FPPS can lead to cardiac hypertrophy, fibrosis, and LV dysfunction by RhoA activation^11^. On the other hand, inhibition of FPPS by alendronate attenuated Ang-II induced cardiac hypertrophy and fibrosis through RhoA^10^. In the present study, GTP-RhoA and geranylgeranylated RhoA were elevated 12 weeks following AAC, while FPPS interfering could not diminish the geranylgeranylated RhoA. However, our previous study found that the GTP-Ras was increased in 3 weeks but activation of RhoA was not altered within 8 weeks after constriction in the Sprague-Dawley rats^12^. Additionally, the altering of FPP firstly affected the synthesis of GGPP, which subsequently affected the geranylgeranylation of RhoA (Fig. 1F). Therefore, the inhibition of active RhoA was not so remarkable as that of Ras in this study. Rac1 and Cdc42 activation were still not varied in the present study. Inhibition of FPPS might also decrease the RhoA activation as time goes on.

In conclusion, we provide here important experimental evidence that inhibition of FPPS improved AAC-induced chronic cardiac remodeling by the reduction of Ras activation. Along with the previous study^10^, inhibition of FPPS effects multiple pathways to improve chronic cardiac remodeling under different stimulus. Inhibitors of FPPS, such as alendronate and zoledronate could be potential therapy in chronic cardiac remodeling. But the association between FPPS and intracellular Ca^2+^ homeostasis still remains unclear. Further studies are required to investigate the different functions of FPPS in acute and chronic heart failure.

## Materials and Methods

### Animals and abdominal aortic constriction

The investigation conformed to the Guide for the Care and Use of Laboratory Animals, published by the US National Institutes of Health (NIH Publication, revised in 2011), and was approved by the Institutional Animal Care and Use Committee of Zhejiang University. The same knockdown of FPPS mouse model was used as previous reported^14^. The transgenic mice were mated with wild-type to keep their offsprings as heterozygote. Third-generation, 3–4 months-old male FPPS transgenic and their non-transgenic littermate control (NLC) were randomly divided into suprarenal abdominal aortic constriction (AAC) or sham operation, as previously described^31^. Briefly, mice were anesthetized by sodium pentobarbital (50 mg/kg, i.p.). The abdominal aorta was constricted at the suprarenal level with 6–0 nylon strings by ligation of the aorta with a blunted 26-gauge needle, which was pulled out thereafter. In the sham-operated group, the ligature was not tightened.

### Echocardiography

12 weeks after operation or sham operation, mice were anaesthetized (1.5% isoflurane). Cardiac function was evaluated using a 14M-HZ 2D guided M-mode echocardiography. Transthoracic two-dimensional and M-mode images were acquired using a Sonos 5500 Echocardiographic 95 System (Philips Medical Systems, Andover, MA, USA) equipped with a transducer (frequency, 14 MHz). M-mode recordings were made from digital images using analysis software (Sonos 5500 software package). M-mode images were used for measurements of interventricular septum wall thickness in end-diastole (IVSd) and left ventricular posterior wall thickness in end-diastole (LVPWd). Fraction shortening (FS) and ejection fraction (EF) were calculated according to measurements of the left ventricular (LV) end-diastolic and end-systolic diameters. Imaging of left ventricular long axis was taken. All measurements were averaged for three consecutive beats.

### Blood pressure

Systolic blood pressure (SBP) was measured 12 weeks after operation or sham operation by the tail-cuff method (Kent Coda 8 Blood Pressure machine, Japan) while the mice were conscious. Data were averaged for 20 consecutive cardiac cycles.

### Histopathology

The day after echocardiographic analysis, mice were sacrificed by cervical dislocation. Hearts were weighed, fixed in 10% formalin for 24 hours and then embedded in paraffin. Sections (5 μm) were stained with hematoxylin and eosin (H&E) or Picrosirius Red staining. The areas of myocardial cell surface and interstitial fibrosis was calculated. Micrographs were taken at low (×4) and high (×40) magnification using fluorescent microscopy. Cardiomyocyte areas were measured in 50 nucleated transverse sections of myocytes in each tissue section, 6 mice per group using ImageJ software (National Institutes of Health, Bethesda, MD, USA). The area of red pixel content was measured as total fibrosis area, and the extent of fibrosis was evaluated by the ratio of fibrosis area to total area. 6 mice per group were measured.

### RNA extraction and quantitative real-time PCR

mRNA expression levels of ANP, BNP, β-MHC, procollangen I and III were analyzed by RT-PCR. Total RNA was isolated from left ventricular myocardium using an RNAiso kit (TaKaRa Bio, Tokyo, Japan). After digestion of the RNA with DNase I (TaKaRa Bio), first-strand cDNA was synthesized by reverse transcription (TaKaRa Bio). PCR was carried out with SYBR^®^ Premix Ex TaqTM (TaKaRa Bio) using 5 μL of cDNA (which corresponded to 20 ng of total RNA in a final volume of 20 μL) and 0.4 μmol/L of each primer. Quantitative PCR was carried out using ABI 7500 (Applied Biosystems, Foster City, CA, USA). Amplification specificity was checked using a melting curve following the manufacturer’s instructions. Data were normalized according to the abundance of GAPDH, and then expressed relative to the mean value for the NTg mice group. The primers were designed as shown in Table 3.

### Western blot analysis

Protein was isolated from the left ventricle. Protein concentrations were measured using a bicinchoninic acid (BCA) Protein Assay Kit (Beyotime Institute of Biotechnology, Haimen, China). An equal volume of 4% Triton X-114 was added to the supernatant to solubilize and fractionate the lipid-rich cell membrane, as previous described^32^. The aqueous upper phase containing enriched intracellular Ras and RhoA, and the organic lower phase, which contained highly enriched membrane-associated Ras and RhoA, was separated by 4% Triton X-114 at 37 °C for 5 min. Each sample underwent electrophoresis on 12% (v/v) sodium dodecyl sulfate-polyacrylamide gel electrophoresis (SDS-PAGE) and transferred to a polyvinylidene difluoride (PVDF) membrane. Membranes were blocked with 5% (w/v) bovine serum albumin in Tween/TBS (TBST) for 1 h at room temperature and incubated with antibody in TBST overnight at 4 °C. Membranes were washed thrice with TBST and incubated with secondary antibody (horseradish peroxidase-conjugated) at room temperature for 1 h. After washing 4–5 times with TBST, proteins were visualized with an enhanced chemiluminescence (ECL) detection reagent (Beit Haemek, Kibbutz Beit Haemek, Israel).

### Activation of RhoA, Rac1, Cdc42 and Ras

Ras, RhoA, Rac1 and Cdc42 activity was determined by an absorbance-based G-LISA Activation Assay Biochemistry kit (Kit ^#^BK131; ^#^BK124; ^#^BK128; ^#^BK127; Cytoskeleton, Denver, CO, USA). Ras activity was determined from protein isolated from left ventricular tissues. The protein was also isolated by homogenizing with cell lysis buffer. This assay used a Ras-GTP-binding protein linked to the wells of a 96-well plate. Active GTP-bound Ras in cell lysates adhered to the wells, whereas inactive GDP-bound Ras was removed during the washing steps. Bound GTP Ras was detected by incubation with a Ras-specific antibody followed by incubation with a secondary antibody conjugated to horseradish peroxidase (HRP) and a detection reagent. The signal was read by measuring absorbance at 490 nm using a Microplate Reader (Model 680, Bio-Rad, Hercules, CA, USA). RhoA, Rac1 and Cdc42 activity were aslo determined similarly according to manufacturer instructions.

### Statistical analyses

Results are the mean ± SEM. ANOVA followed by the multiple-comparison Bonferroni t-test was used to determine significant differences among 4 groups. SPSS version 19.0 (SPSS, Chicago, USA) was used for all analyses. P < 0.05 was considered statistically significant.

## Additional Information

**How to cite this article**: Zhao, C.-Z. *et al*. Inhibition of farnesyl pyrophosphate synthase improves pressure overload induced chronic cardiac remodeling. *Sci. Rep.* **6**, 39186; doi: 10.1038/srep39186 (2016).

**Publisher's note:** Springer Nature remains neutral with regard to jurisdictional claims in published maps and institutional affiliations.

## Figures and Tables

**Table 1:** Organ weights and blood pressure in NLC and transgenic FPPS mice 12 weeks after AAC or SHAM.

	NLC-SHAM (n = 10)	NLC-AAC (n = 10)	Tg-SHAM (n = 10)	Tg-AAC (n = 10)
Body weight (g)	29.6 ± 0.8	30.5 ± 0.8	29.9 ± 0.4	30.8 ± 0.6
Heart weight (g)	123 ± 5	154 ± 5***	122 ± 2	141 ± 3**
Heart weight/body weight (mg/g)	4.13 ± 0.08	5.04 ± 0.10***	4.08 ± 0.07	4.58 ± 0.06***^,##^
Heart weight/tibia length (mg/mm)	5.22 ± 0.17	6.67 ± 0.18***	5.23 ± 0.09	5.97 ± 0.11**^,##^
Lung weight (g)	160 ± 5	178 ± 7	157 ± 4	171 ± 8
Lung weight/Body weight (mg/g)	5.38 ± 0.11	5.62 ± 0.16	5.24 ± 0.14	5.52 ± 0.17
Systolic blood pressure of tail artery (mmHg)	119.3 ± 4.0	123.1 ± 4.5	118.4 ± 4.1	120.5 ± 4.0

Data are mean ± SEM. NLC, non-transgenic littermate control; Tg, transgenic; FPPS, farnesyl pyrophosphate synthase; AAC, abdominal aortic constriction; ***P < 0.001 vs. matched SHAM; **P < 0.01 vs. matched SHAM; ^##^P < 0.01 vs. NLC-AAC; ^#^P < 0.05 vs. NLC-AAC.

**Table 2:** Echocardiographic assessment of cardiac structure and function in NLC and transgenic FPPS mice 12 weeks after AAC or SHAM.

	NLC-SHAM (n = 10)	NLC-AAC (n = 10)	Tg-SHAM (n = 10)	Tg-AAC (n = 10)
IVSd (mm)	0.83 ± 0.03	1.02 ± 0.04***	0.82 ± 0.02	0.97 ± 0.02**
LVPWd (mm)	0.83 ± 0.02	0.98 ± 0.04*	0.82 ± 0.02	1.01 ± 0.05**
LVIDd (mm)	3.16 ± 0.14	4.32 ± 0.20***	3.10 ± 0.10	3.71 ± 0.10*^,#^
LVIDs (mm)	2.02 ± 0.08	3.25 ± 0.16***	1.98 ± 0.08	2.59 ± 0.08**^,##^
Ejection fraction (%)	73.6 ± 1.1	56.8 ± 1.8***	73.5 ± 1.9	65.6 ± 1.2**^,##^
Fractional shortening (%)	36.0 ± 0.9	24.5 ± 1.0***	36.1 ± 1.5	30.0 ± 0.8**^,##^
Heart Rates (beats/min)	532 ± 18	525 ± 16	518 ± 19	543 ± 21

Data are mean ± SEM. NLC, non-transgenic littermate control; Tg, transgenic; FPPS, farnesyl pyrophosphate synthase; AAC, abdominal aortic constriction; IVSd, interventricular septum thickness in end-diastole; LVPWd, left ventricular posterior wall thickness in end-diastole; LVIDd, left ventricular internal dimension in end-diastole; LVIDs, left ventricular internal dimension in end-systole; ***P < 0.001 vs. matched Sham; **P < 0.01 vs. matched SHAM; *P < 0.05 vs. matched SHAM; ^#^P < 0.05 vs. NLC-AAC; ^##^P < 0.01 vs. NLC-AAC.

**Table 3:** The sequences of primers for quantitative real-time PCR.

Target gene	Sequence
Mouse-ANP	F: 5- CTCCATCACCCTGGGCTTCTTCCTCGTCTT -3′
	R: 5′- GGTGGTCTAGCAGGTTCTTGAAATCCATCA -3′
Mouse-BNP	F: 5- GTCAGTCGTTTGGGCTGTAAC -3′
	R: 5′-AGCGGACTGGGCTGTAACAG-3′
Mouse-β-MHC	F: 5- GTCAGTCGTTTGGGCTGTAAC -3′
	R: 5′- CGCATAATCGTAGGGGTTGT -3′
Procollagen I	F: 5′- GAGCGGAGAGTACTGGATCG-3′
	R: 5′- GCTTCTTTTCCTTGGGGTTC-3′
Procollagen III	F: 5′- GTCCACGAGGTGACAAAGGT-3′
	R: 5′- GATGCCCACTTGT TCCATCT-3′
Mouse-GAPDH	F: 5′- TGTGTCCGTCGTGGATCTGA-3′
	R: 5′- TTGCTGTTGAAGTCGCAGGAG-3′
